# Co-Operative Appeals

**Published:** 1918-11-30

**Authors:** 


*To the Editor of* THE HOSPITAL.

### Co-operative Appeals.

*To the Editor of* THE HOSPITAL.

SIR,—Six of the London hospitals are taking part in a scheme for collecting funds with the aid of the *Gentlewoman*. The last plan of this sort was a Mansion House fund on behalf of seven of the London orphanages, when a sum of between £6,000 and £7,000 was raised. The present plan is styled "A Costless Thankoffering," which, as it stands, is not a very happy title. Nothing valuable, for instance, is done in the begging line without cost of brain. I hope the six hospitals concerned will reap a good reward, but am inclined to believe that individual effort pays best in these matters.

It pays an institution to preserve its own personality. This "mixed" idea may justify itself—I trust it may—but I have my doubts, and strongly incline to single effort and enterprise. As a rule, one is told that "two heads are better than one," but in this case it seems to be taken for granted that one head is better than six, for it is the Editor of the *Gentlewoman* who is reported to have devised the scheme.—Yours faithfully,

HOSPITAL SECRETARY.

[While co-operative subscriptions through the great funds and kindred organisations hold the field, co-operative appeals are still a matter of controversy. Our correspondent expresses his disbelief in them. It rests with those in favour to confront him with their arguments, unless he is to have the last word.—ED. THE HOSPITAL.]

